# Synthesis and Sensing Performance of Chitin Fiber/MoS_2_ Composites

**DOI:** 10.3390/nano13091567

**Published:** 2023-05-06

**Authors:** Yuzhi Zhang, Zhaofeng Wu, Jun Sun, Qihua Sun, Fengjuan Chen, Min Zhang, Haiming Duan

**Affiliations:** 1 School of Physics Science and Technology, Xinjiang University, Urumqi 830046, China; 2 Xinjiang Key Laboratory of Solid State Physics and Devices, Xinjiang University, Urumqi 830046, China

**Keywords:** MoS_2_–CFs composites, chitin fibers, hydrothermal method, gas-sensitive sensors, wearable strain sensor, strain-sensitive

## Abstract

In this study, chitin fibers (CFs) were combined with molybdenum sulfide (MoS_2_) to develop high-performance sensors, and chitin carbon materials were innovatively introduced into the application of gas sensing. MoS_2_/CFs composites were synthesized via a one-step hydrothermal method. The surface properties of the composites were greatly improved, and the fire resistance effect was remarkable compared with that of the chitin monomer. In the gas-sensitive performance test, the overall performance of the MoS_2_/CFs composite was more than three times better than that of the MoS_2_ monomer and showed excellent long-term stability, with less than 10% performance degradation in three months. Extending to the field of strain sensing, MoS_2_/CFs composites can realize real-time signal conversion in tensile and motion performance tests, which can help inspectors make analytical judgments in response to the analysis results. The extensive application of sensing materials in more fields is expected to be further developed. Based on the recycling of waste chitin textile materials, this paper expands the potential applications of chitin materials in the fields of gas monitoring, biomedicine, behavioral discrimination and intelligent monitoring.

## 1. Introduction

With the advancement of industrialization and the proliferation of population, the problem of air pollution has gradually become an important aspect of development that cannot be ignored. Polluting gases can expand in scope as the atmosphere moves, causing near irreversible damage to the atmosphere that, when inhaled by humans, can damage the human brain by impairing cognitive abilities [1,2]. Every year, millions of people die from inhaling harmful gases, and the World Health Organization has issued warnings about air quality.

In this context, air quality monitoring is particularly important. Whether in industrial, agricultural, military, medical or daily household sectors, perfect gas monitoring can strongly guarantee the normal operation of production or daily life. Gas sensors are important gas monitoring devices that have emerged in various fields and have made great contributions to the monitoring of the gas environment [3,4,5]. The core part of gas sensors is the sensing material, which is used to determine the type and concentration of gases by identifying the differences in the target gases and the specific response changes in electrical conductivity, which, in turn, conducts signals. At present, metal oxides are often used as the sensing part of sensitive materials [6,7], and have shown a good response performance and economy. However, with changes in the application scenarios and improvements in the air environment monitoring requirements, some problems are emerging [8,9,10]. These include the fact that the material stability needs to be further strengthened and that, when monitoring flammable and explosive gases, there may be certain hidden dangers that mean safety cannot be absolutely guaranteed. At the same time, compared with some biomass materials, the preparation process of metal oxide materials is more cumbersome. Two-dimensional materials have gradually revealed their advantages and are now widely used in many fields in systems that involve two-dimensional materials, surface properties, electrical properties and functional properties [11,12]. In addition, they have become a great area of interest in gas-sensitive materials when their excellent semiconductor properties are combined with biomass materials [13,14,15,16,17,18].

Similar to gas-sensitive sensors, the application of strain sensing is also based on conversion between signals [19,20,21]. Under a constant voltage, the material does not deform and outputs a stable current. After the force, the current fluctuates according to the degree of force, which is reflected in the signal changes. When the material is stimulated by an external force, the conductivity of the material changes, which is reflected in the electrical signal, and the value is output, according to the magnitude of the value; thus the deformation state of the monitored object can be discriminated. Flexible electronics are usually artificial wearable devices with sensing capabilities [22,23,24,25,26] like those of human skin, that are based on mechanical, optical, and air-sensitive sensing principles; these devices are capable of monitoring external stimuli, such as pressure, strain, temperature, or humidity, and converting them into visible electrical signals, and have great applicative potential regarding the human–machine interface and personalized health monitoring. The key challenge for wearable electronics is the organic combination of superior mechanical flexibility and keen sensory capabilities, while being more adaptable to biologically attached wear, which requires device materials that are biologically non-toxic and non-harmful within the context of good mechanical and electrical properties. At present, the practical application of flexible electronic devices in the fields of health monitoring, auxiliary medicine, and patient rehabilitation has achieved remarkable results. For hearing-impaired people, visual or tactile signals can be used to compensate for hearing deficiencies, and for injured patients or the elderly, their activities or behavioral states can be identified and monitored based on real-time signals. However, further basic theoretical research and in-depth experimental testing are needed in order to realize these devices’ more extensive application, such as in flexible devices [27,28,29,30,31,32,33].

At present, a large number of relevant studies have demonstrated the performance and practical applications of biomass materials in the field of sensing [34,35,36,37,38], incorporating and building in different systems to exploit their material properties. Carbon materials obtained via the conversion of biomass materials, such as carbon fibers, also have a wide range of applications, and have excellent physicochemical and electrical properties [39,40,41]; the potential of biomass carbon materials in other sensing fields is gradually being realized, such as in the field of strain sensing, where the sensing material changes its electrical conductivity according to the deformation, and the signal conversion between mechanics and electricity is performed through the reception of external stimuli. The construction of flexible electronic devices that select and process flexible substrates is indispensable, and the structural and functional properties of biomass materials enable them to become the core materials of sensor devices [42,43,44].

Chitin, as a natural polysaccharide with high reserves on earth, is a biomass material with great potential and has been widely developed and applied due to its excellent physicochemical properties [45,46,47]. Kyungtae Kim et al. obtained a high-performance biodegradable chitin polymer from squid bone material [48] and demonstrated its usefulness as a flexible piezoelectric material. The easily controlled ferroelectric chitin film exhibited excellent piezoelectricity properties under external mechanical pressure and its performance was comparable to that of conventional fluorine-based piezoelectric polymers. In addition, the biodegradable chitin polymer could be successfully dissolved by chitinase within 8 days without any toxic residue. Liu XJ et al. developed a colorimetric sensor for detecting the degree of food oxidation [49], which was prepared by using a chitin nanowhisker film that combines a dye and hydroxylamine sulfate. The actual tested sensing performance was excellent and showed good selectivity, and was also less susceptible to interference from other substances. Rafaela et al. developed a disposable ethanol sensor [50] that was prepared using a composite of chitin and metal materials. The ethanol sensor constructed using this composite exhibited high sensitivity at room temperature. Pejman et al. developed a hydrogel with excellent electrical conductivity [51] that was made by using a composite of chitin as the main material. The hydrogel also showed excellent mechanical properties, a stable structure and self-healing properties. This work taps into the potential of chitin materials in biomedical and mechanical applications. According to the research, chitin has been widely used in various fields due to its physical and chemical properties, but the carbon materials that sacrifice the chitin template have not been fully used, which is also the focus of this work.

Among chitin and its derivatives, spun chitin fibers have good air permeability and a high specific surface area, as well as good biocompatibility, and have been used in a large number of medical materials and apparel [52,53]. As a metal sulfide two-dimensional material, molybdenum disulfide (MoS_2_) has a layer structure similar to that of graphite and is maintained by relatively weak van der Waals forces between the layers, exhibiting many excellent properties [54,55]. In this work, for the recycling of waste chitin fiber (CF) materials and the functional development of chitin materials, a one-step hydrothermal method was used to prepare MoS_2_, hydrothermal chitin fibers (H-CFs), and chitin molybdenum disulfide composite material (MoS_2_/CFs); these materials were prepared in order to extend the application of chitin materials in the field of gas sensing and that of sensing properties in other fields, in combination with MoS_2_ two-dimensional materials, and to avoid the high energy consumption problem encountered during the MoS_2_ synthesis process. Furthermore, the MoS_2_/CFs produced have shown excellent performance in both gas-sensitive and strain sensing fields after performance tests. The study provides an idea for the utilization of waste chitin products, helps to alleviate the problem of the energy-extensive consumption of MoS_2_ preparation, and promotes the development of chitin materials in the field of gas sensing and mechanical sensing applications.

## 2. Materials and Methods

### 2.1. Materials

Ammonium molybdate ((NH_4_)_6_Mo_7_O_24_·4H_2_O), thiourea (CH_4_N_2_S), ethanol (C_2_H_6_O), formaldehyde (CH_2_O), hydrogen peroxide (H_2_O_2_) and ammonia monohydrate (NH_3_·H_2_O, 25–28%) were used as the analytical reagents and came from Sinopharm Chemical Reagent Co. The chitin textile fiber was purchased from Qingdao Yunzhou Technology Co., Qingdao, China.

### 2.2. Preparation of MoS_2_,H-CFs and MoS_2_/CFs

**Figure 1:**
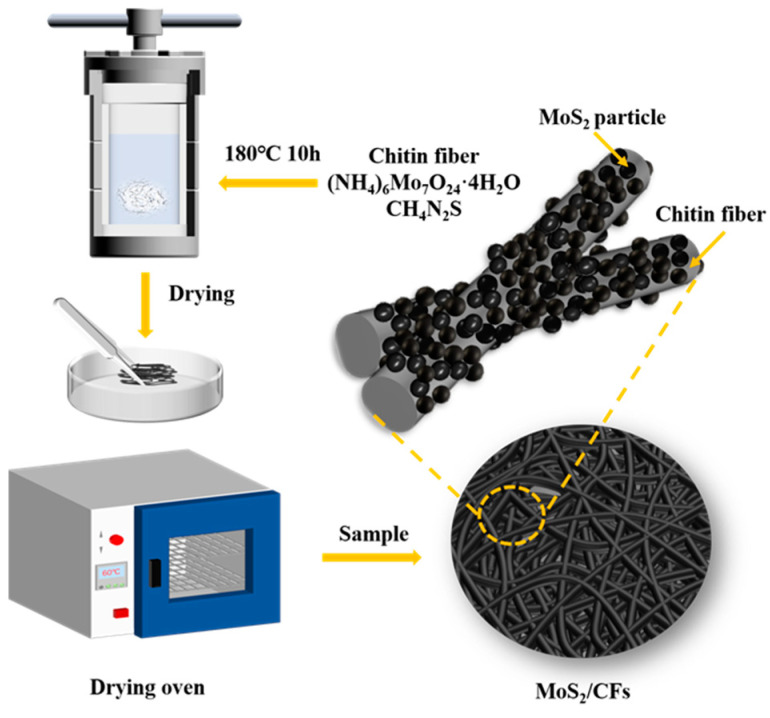
Schematic diagram of the process of synthesizing MoS_2_/CFs composites via a hydrothermal reaction and drying.

MoS_2_ and MoS_2_/CFs were prepared via a one-step hydrothermal method combining ammonium molybdate and thiourea using spun chitin fibers as raw materials (Figure 1). Then, (NH_4_)_6_Mo_7_O_24_·4H_2_O (1 mmol) and CH_4_N_2_S (30 mmol) were added to 35mL of deionized water. After using a magnetic mixer for 30 min to fully dissolve the material at room temperature, the chitin fibers with a homogeneous texture were added to the solution. Then, the solution was transferred into 50 mL of polytetrafluoroethylene and prepared for the hydrothermal reaction. The reaction kettle was sealed, the starting temperature was set to 60 °C, and the heating/cooling rate was set to 2 °C/min. After the reaction reached 180 °C, heat preservation was carried out for 6 h. Once this had been completed, the reaction kettle was lowered to room temperature and taken out. The material was removed and washed in deionized water three times, and then dried in a drying oven at 60 °C for 8 h. In this manner, MoS_2_/CFs were obtained. The black precipitate obtained was washed and dried in the same way in order to obtain a MoS_2_ powder. In addition, a blank hydrothermal control material (H-CFs), without the addition of ammonium molybdate and thiourea, was prepared under the same conditions.

### 2.3. Device Fabrication and Testing

#### 2.3.1. Gas Sensing Performance Testing

An appropriate amount of sensing material was ground and deionized water was added dropwise. This was mixed thoroughly into a paste and coated on the electrode; the length of the coated area of the electrode sheet was 7 mm and the width was 3 mm. The electrode was dried at room temperature, connected to an electrochemical workstation (CHI660E, Chenhua, Shanghai, China), and aged at 4 V for 24 h to obtain a stable sensing chip. The vapor of the gas to be measured was derived using thermal evaporation, according to Equation (1):

Q = (V × C × MW)/(22.4 × d × ρ) × 10^−9^ × (273 + T_R_)/(273 + T_C_) (1)

where Q is the volume of the liquid taken; V represents the volume of the vessel in which the test was performed; MW is the molecular weight of the selected substance; D represents the purity of the selected liquid; C represents the concentration of the target gas; ρ is the density of the liquid; T_R_ is the room ambient temperature; and T_C_ is the temperature inside the test vessel. The sensing signals were recorded at room temperature using a photoelectric gas-integrated test rig (CGS-MT, Zhongju High-tech, Beijing, China). In the test, a voltage of 4 v was applied to both ends of the sensing chip. The response was defined as follows:

(2) $$\mathrm{Response}=\left(\frac{I_{G}-I_{R}}{I_{R}}\right)\times 100\%$$

where I_R_ and I_G_ are the current output in the reference gas and the target gas, respectively. The test standards were determined by referring to the industry standard for planar thick-film semiconductor gas sensing devices (JB/T 11623-2013): the response time and recovery time were defined as 90% and 10% of the maximum value of the contact reached between the sensing material and the gas to be measured. The formula for calculating the standard deviation of the response value was as follows:

(3) $$S=\sqrt{\left({\left(\sum (xi-\bar{x}\right)}^{2})/(n-1)\right)}$$

where S represents standard deviation, xi represents each response value in the data, $\bar{x}$ represents the mean value of the data, and n represents the number of data.

#### 2.3.2. Strain Sensing Performance Testing

The conductivity (σ, S/cm) of the sensing material was measured and analyzed by using the (T&H, Shanghai, China) electrochemical workstation CHI660E.

σ = L/(R × A),(4)

where L represents the distance between two adjacent probes, A is the cross-sectional area of the sensing material, and R is the resistance output value [56,57]. The sensor material with uniform texture was taken, and then the flexible substrate was vacuum-encapsulated with polyethylene material to construct the sensor. The flexible electronic integrated test platform (AES-4SD) kept the sensor in a naturally unstressed state. The ends of the sensor were clamped with silver electrodes and fixed with screws on both sides, so that the components did not loosen on the platform. Then, stress tests were conducted. The single cycle was set as 3, the tensile stress was increased by 10% step by step, and the input voltage was 4000 mV. The test started after the component was pre-stretched. The bending stress of the sensor was tested similarly. Once the bending had been obtained, a fixed amount of force was applied perpendicular to the bending tangent of the material. Then the signal was tested in the same way.

During the strain sensing signal testing, the sensing capability of monitoring human motion was also studied by using a flexible electronic integrated test platform (AES-4SD). Flexible tape was applied to the detection area to maintain the level of adhesion and force between the sensor and the area. Then, 4 V was applied at both ends of the sensing element, pressure was applied to the sensing material, and strain was generated at room temperature. Changes in electrical signals in the sensing material were recorded by the test platform, and the response was defined as follows:

(R − R_0_)/R_0_ × 100%, (5)

where R_0_ and R are the sensing material resistance output values before and after the deformation.

## 3. Results

### 3.1. Surface Characterization and Structural Analysis of MoS_2_/CFs

**Figure 2:**
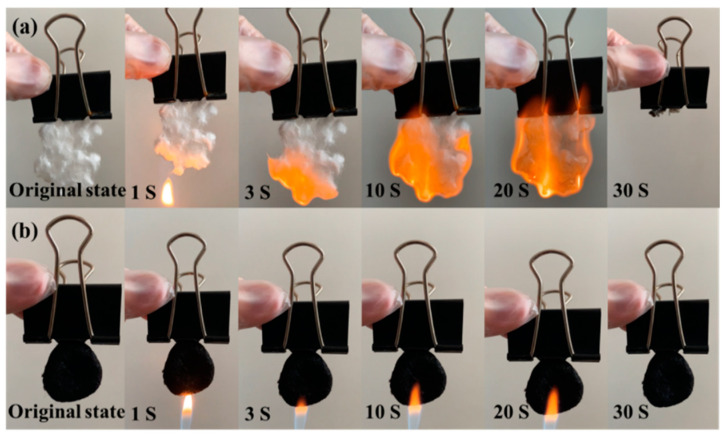
(**a**) CFs, (**b**) MoS_2_/CFs ignition and continuous supply of butane material flame retardant test.

As shown in Figure 2, the experimentally prepared MoS_2_/CFs and the CFs that had been uniformly dispersed were jointly burned by flame and continuously supplied with butane gas, and the CFs were partially burned in the flame, which was accompanied by melting; meanwhile, the MoS_2_/CFs were not ignited in the open flame and did not melt and deform, which shows that the prepared composites had certain fire resistance properties compared with the raw materials.

**Figure 3:**
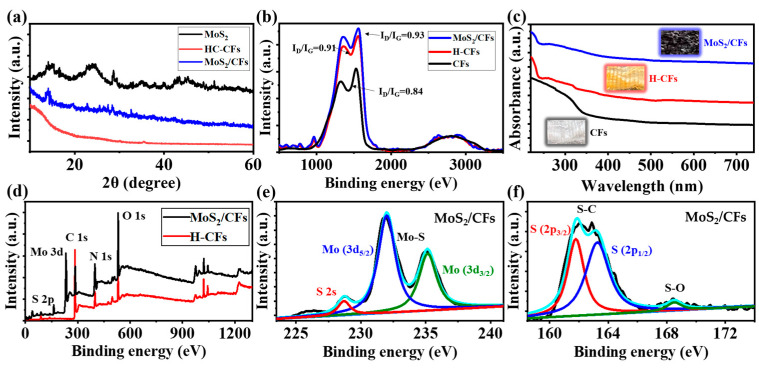
(**a**) XRD patterns of MoS_2_, H-CFs and MoS_2_/CFs; (**b**) Raman spectra; (**c**) UV–Vis patterns of CFs, H-CFs and MoS_2_/CFs, and XPS spectra of MoS_2_/CFs composites and H-CFs; (**d**) high-resolution spectra, (**e**) Mo3d, and (**f**) S2p.

The MoS_2_ powder prepared in the experiment was characterized using XRD (Figure S1 in Supplementary Materials), which corresponded to the line of the standard XRD JPCDS card. As shown in Figure 3a, when comparing the three curves in the XRD pattern with the analysis of Figure S2, it is obvious that the characteristic peak of chitin fibers in the H-CFs curve is weakened and transformed into a broader graphite peak under hydrothermal carbonization. As shown by the XRD curve of MoS_2_/CFs, an obvious characteristic peak is produced at 2θ = 14.4°, corresponding to the (002) crystal plane of hexagonal MoS_2_, which at 2θ = 32.3° produced a low-frequency diffraction peak, which was caused by the (100) face in 2H-MoS_2_ of the loaded material [58,59]; this tentatively demonstrates the successful preparation of MoS_2_/CFs. The structural changes, degree of graphitization and disorder in the materials were characterized via Raman spectroscopy. As shown in Figure 3b, both H-CFs and MoS_2_/CFs exhibit distinct carbon material characteristics in the G and D bands. By observing the curves in the Raman spectra, through the calculation of the peak height, the I_D_/I_G_ of the CFs, H-CFs, and MoS_2_/CFs materials are 0.84, 0.91, and 0.93, respectively. To some extent, Raman spectroscopy reflects the change in the material structure, from biomass materials to carbon materials with highly defective structures and semiconductor properties. After hydrothermal carbonization [60], the defect degree of the material increases to some extent, while the MoS_2_/CFs have the highest defect degree, and the structure of the material is reflected in the specific sensing properties. In addition, observing Figure 3c, the UV–visible spectra of the materials also differed significantly from each other [61], and the highest absorbance of MoS_2_/CFs further proved the successful synthesis of the composites, while the successful loading of MoS_2_ on the chitin fibers also promoted the flame retardant and heat resistant properties of MoS_2_/CFs; it is these that provided the basis for the surface structural properties of the materials.

To determine the types of elements that the composite contained, the chemical states of the MoS_2_/CFs composite were investigated via XPS. As shown in Figure 3d, the main constituent elements in the MoS_2_/CFs composites were S, Mo, C, N and O, with total element percentages of 14.22%, 12.83%, 33.51%, 9.32%, and 30.12%. In contrast, H-the CFs contained three elements, C, N and O, in total element percentages of 77.04%, 11.1%, and 11.86%, respectively. In the MoS_2_/CFs composite, the occurrence of S and Mo elements came from the loading of MoS_2_. It can be seen in Figure 3e that two peaks of Mo 3D appeared at the positions of 232.1 and 235.4 eV. Figure 3f shows the S 2p peak of the MoS_2_/CFs; the two peaks at 161.2 and 163.8 eV reflected the S–C functional group and the peak of 168.4 eV the was S–O functional group. Compared with H-CFs, in MoS_2_/CFs composites, the ratio of C elements to O elements is significantly lower, which laterally responds to the increased carbonization of the material in the hydrothermal reaction under the condition that the outer layer is not loaded with MoS_2_, and this leads to a further increase in the electrical conductivity of the material. In MoS_2_/CFs composites, the ratio of the elements is balanced and the carbonization level is appropriate, which may be one of the factors that affects the subsequent gas-sensitive performance of the material.

**Figure 4:**
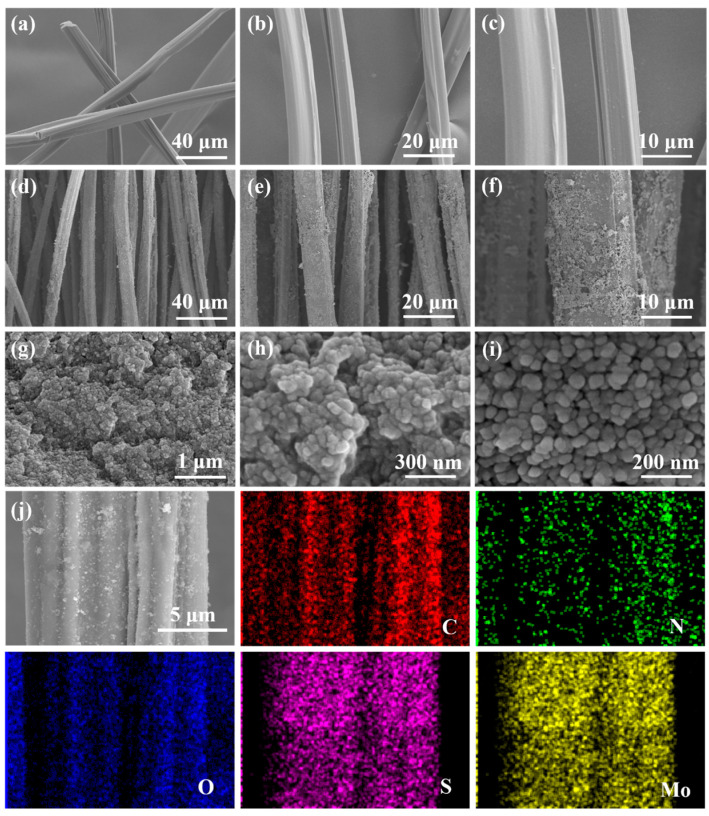
SEM patterns of (**a**–**c**) H-CFs, (**d**–**f**) MoS_2_/CFs, (**g**–**i**) MoS_2_ powder. (**j**) Elemental distribution of MoS_2_/CFs composites.

As shown in Figure 4a–f, the size of the chitin fibers did not change, but it is more obvious that the fiber surface turned from smooth to rough, covering a large area of MoS_2_ sensing material, and that the uniformly grown MoS_2_ isolated the outer surface of the fiber to a certain extent [62,63,64,65] and played a full encapsulation role; this facilitated the realization of the flame retardant effect, and at the same time, the sensing performance of the MoS_2_/CFs was made to act as a structural pavement for the sensing performance of the MoS_2_/CFs. Figure 4g–i shows the experimentally prepared MoS_2_ powder on the CFs, which shows an overall nanoflower state; however, limited by the particle size, the MoS_2_ powder material showed a large amount of agglomeration when a low surface energy was sought for. Despite the influence of surface energy, when relying on the large size of CFs, with an average diameter of more than 10 microns, the nanoscale MoS_2_ still had a relatively uniform distribution on CFs, showing an inclination for encapsulation. To further determine the distribution of different elements on the material, the composites were subjected to elemental analysis tests. As shown in Figure 4j, in addition to the C, N, and O elements possessed by chitin itself, Mo elements and S elements were also uniformly loaded on top of the chitin fibers. In combination with Table S1, it is evident that the proportions of each element are basically consistent with the results of XPS. The EDS mapping image shows a clear and uniform distribution of light and dark on the images. From the images (Figure 4j), it can be proven that various elements have uniform distributions on the composite materials.

### 3.2. Analysis of Sensing Performance

**Figure 5:**
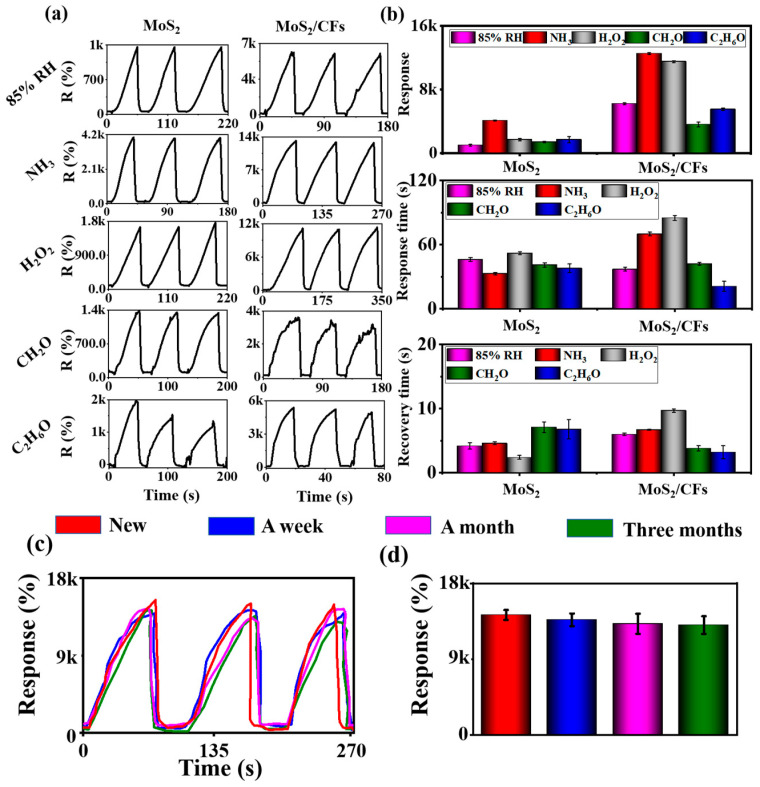
(**a**) Gas-sensitive response of sensors prepared using MoS_2_ and MoS_2_/CFs; (**b**) comparison of response size, response time and recovery time of MoS_2_ and MoS_2_/CFs; (**c**,**d**) long-term stability test of MoS_2_/CFs to 1000 ppm NH_3_.

The prepared MoS_2_ powder and MoS_2_/CFs were used for electrode sheet fabrication and gas-sensitive performance testing. As shown in Figure 5a, the overall response of the MoS_2_/CFs to the five target gases (85% RH, NH_3_, H_2_O_2_, CH_2_O, C_2_H_6_O) was more than three times better than that of the MoS_2_ powder, and their response to the specific gases was also better. For the MoS_2_ monomer, the presence of the agglomeration phenomenon led to a significant reduction in the specific surface area of the material, which inevitably affected the gas-sensitive monitoring and reduced the contact area with the target gas during the detection process. In the case of MoS_2_/CFs, the chitin fibers provided better attachment sites for MoS_2_ [62,63,64,65] and reduced the agglomeration phenomenon, thus allowing better contact with the target gas molecules during the gas-sensitive testing. Meanwhile, an observation of the response time and recovery time reveals that the response time of the composites increased slightly but to a lesser extent, probably due to the denser distribution of the loadings and the greater contact distance of the gas molecules, while the overall change in the recovery time was small and negligible (Figure 5b).

For the practical application of the sensor, the repeatability and long-term stability appear to be crucial. To evaluate the performance of the sensor in practical applications, the stability and repeatability of the MoS_2_/CFs were tested five times in three consecutive months (Figure 5c,d), and its response curve to 1000 ppm of NH_3_ vapor was recorded; the results showed that compared to the newly prepared sensor, the performance degradation did not exceed 10% after three months, and the observation of the response curves showed that the sensor exhibited good reproducibility and long-term stability, and thus the potential to be applied in the monitoring of practical scenarios.

**Figure 6:**
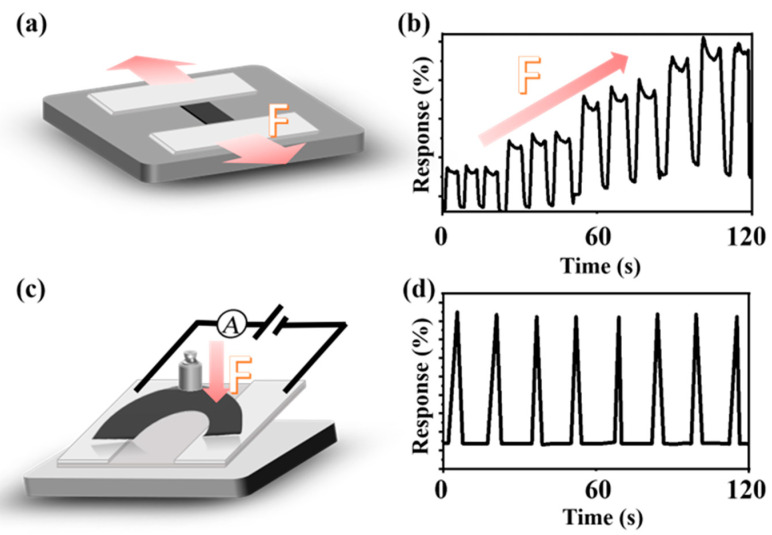
(**a**) Schematic diagram of material stretching, (**b**) change in current signal flowing inside the material for increasing stretching force, (**c**) schematic diagram of applying a fixed force to the bent material, (**d**) change in current signal flowing inside the material.

The performance of MoS_2_/CFs as gas-sensitive sensing materials is significantly enhanced compared to monomers, and MoS_2_, as a widely used material in the sensing field, has also been studied for its electrical properties. After gas-sensitive testing, it was decided that the sensing properties of the prepared composites would be extended to mechanics and that relevant tests would be conducted. When the material was in the tensile state (Figure 6a), the electric signal reflected by the current flowing through the material showed an obvious pattern alongisde the increase in the tensile force. (Figure 6b). The output signal was also found to be clear and stable when the current signal through the bent MoS_2_/CFs composite was examined with a fixed force (Figure 6c,d). The electrical signal output inside the material can reflect the force applied to it more clearly, and the signal transmission can be completed almost instantaneously when the force is applied, thus realizing the process of converting the mechanical signal into an electrical signal.

**Figure 7:**
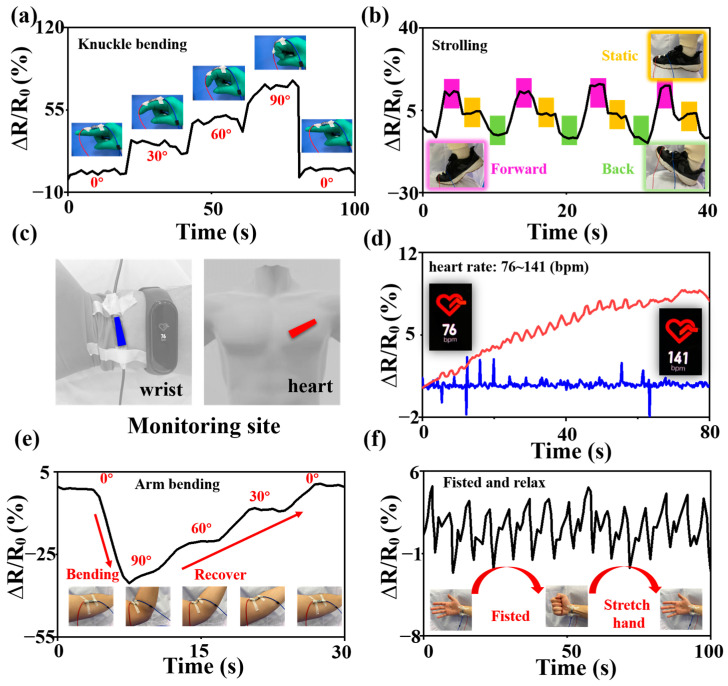
Resistance variation response of MoS_2_/CFs in (**a**) finger joint bending action test, (**b**) simulated walking action test, (**c**) sensor attachment site, (**d**) heart rate change and breathing action test, (**e**) elbow joint bending action test, (**f**) fist clenching action test.

When the sensing material is fixed in a specific position on the skin, the sensing material will be squeezed from different directions with the stretching and contraction of the human epidermis, and the electrical signal of the material will be collected at this time. The collected electrical signal will alter with the change in the object’s motion state, as shown in Figure 7. In turn, the object’s motion state is judged, and the overall state is monitored to distinguish the different movements of the fingers, elbows, ankles, and wrists, for example. In this study, the deeper heart rate was also explored (Figure 7c,d), and it was found that compared to the wrist area, the heart area produces significant electrical signal fluctuations when the heart rate rises, as the respiratory rate accelerates and the chest cavity undulates. In the construction of a modern IoT system, one of the core points is to be able to achieve connectivity and controllability between objects [66,67]. Within such a context, it is crucial that the state of objects in the environment are discriminated and identified; as such, the above strain sensing test not only adds a new path for research in the field of medical rehabilitation, but also provides new ideas for the selection of sensing materials for the construction of IoT facilities.

### 3.3. Analysis of Gas-Sensitive Sensing Mechanism

**Figure 8:**
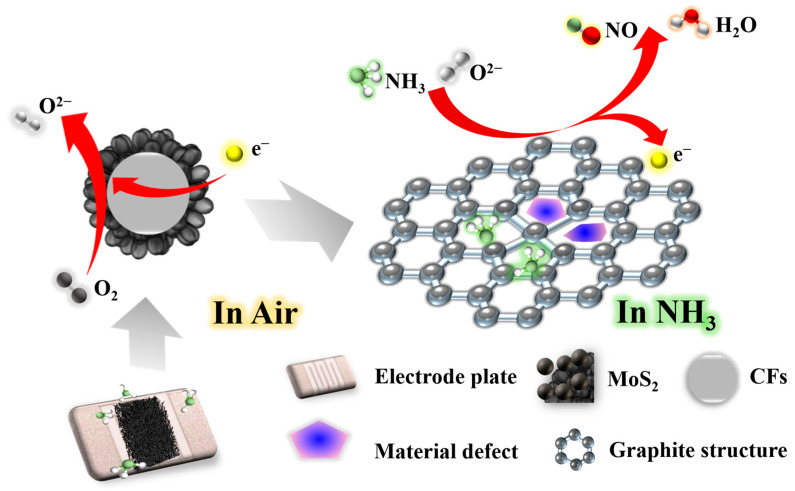
Gas-sensitive response sensing mechanism analysis of MoS_2_/CFs sensors in different atmospheres.

As shown in Equation (6) [68,69,70], for the performance of the chemo-resistive gas sensor, two main considerations are the charge depletion layer (*L*) and the structure of the sensitive material; in the latter, the depth of the depletion layer is proportional to the oxygen ion concentration ($N_{t}$) on the surface of the sensing material and inversely proportional to the carrier concentration ($N_{d}$) of the sensing material [71], and the sensitivity of the sensing material increases with the increase in the depth of the depletion layer when the material structure is the same. Therefore, the sensitivity can be improved by increasing the concentration of oxygen ions or decreasing the density of carriers. In the air environment (Figure 8), oxygen molecules can capture electrons on the material surface at room temperature, thus converting them into chemisorbed oxygen anions s (O^2−^):

(6) $$L\propto \sqrt{\frac{N_{t}^{2}}{N_{d}^{2}}}=\frac{N_{t}}{N_{d}}$$

O_2_ (gas) → O^2−^(ads) (7)

O_2_ (ads) + e^−^→O^2−^(ads) (8)

Meanwhile, a certain L thickness is formed on the material’s surface, which raises the resistance. In the NH_3_ atmosphere (Figure 8), the following reactions occur on the material surface [72]:

4NH_3_(gas) + 5O^2−^(ads) → 4NO + 6H_2_O + 5e^−^ (9)

At the same time, electrons are released to the material surface, and the sparse structure of the CFs’ material surface, surface defects, and MoS_2_ doping also have an important effect on the gas-sensitive performance. Compared with the MoS_2_ monomer, the composite material has more advantages with regard to improving the response size of the target gas without prolonging the response time and recovery time, and the high defect degree possessed by the material also enables the existence of more adsorption sites for gas molecules, which further improves the response to NH_3_.

## 4. Conclusions

Based on the recycling of waste chitin fiber products and the development of the sensing properties of chitin materials, chitin fiber and molybdenum sulfide composites (MoS_2_/CFs) were successfully prepared via a one-step hydrothermal method; MoS_2_/CFs showed excellent surface properties and greatly improved the flame retardancy, while effectively avoiding the large agglomeration of the MoS_2_ monomer to some extent. The sensing performance of the MoS_2_/CFs was further tested, and in terms of gas-sensitive sensing, the overall performance of the MoS_2_/CFs for specific gases was improved by more than three times compared to that of the monomer, showing a better practical application effect. Combined with its long-term stability, the response degree reduction after three months was no more than 10%, showing that the MoS_2_/CFs composite has the potential to be practically applied in various environments with differing gas detection needs. In addition, MoS_2_/CFs showed excellent performance in strain sensing tests, converting strain signals into electrical signals in real time in tensile and motion performance tests, and monitoring and judging the motion of the monitored objects in real time. The sensing materials are expected to be further developed and made into overall flexible electronic devices for medical rehabilitation, bio-intelligence, and Internet of Things development and construction, in order to realize the monitoring of objects’ movement status in different environments. In the study, chitin-based carbon materials have been applied to gas sensing for the first time and the defined experimental process provides a method by which to regulate the gas-sensitive properties of chitin-based materials. Extending their study to the field of strain sensing will further broaden its application in the field of sensing materials. This study provides a reference for the recycling of biomass waste and the development of high performance and low-budget gas sensors.

## Data Availability

All data included in this study are available upon request by contact with the corresponding author.

## Supplementary Materials

The following supporting information can be downloaded at: https://www.mdpi.com/article/10.3390/nano13091567/s1, Figure S1. XRD patterns of MoS_2_ with standard XRD JPCDS card of MoS_2_. Figure S2. XRD patterns of CFs and H-CFs. Table S1. Scanning analysis of C, N, O, S and Mo elements in EDS-Mapping.
