# Supplementary material for: Synthesis and Sensing Performance of Chitin Fiber/MoS2 Composites
Source: Nanomaterials (Basel). 2023 May 6;13(9):1567. doi: 10.3390/nano13091567 (PMC10180960; doi:10.3390/nano13091567)
Supplement: Supplementary file 1 [file nanomaterials-13-01567-s001.zip › nanomaterials-2355538-supplementary.pdf]

Supplementary Materials

Synthesis and Sensing Performance of Chitin Fiber/MoS_2 Composites

Yuzhi Zhang [1,2], Zhaofeng Wu [1,2,*], Jun Sun [1,2,*], Qihua Sun [1,2], Fengjuan Chen [1,2], Min Zhang [1,2] and Haiming Duan [1,2]

[1] School of Physics Science and Technology, Xinjiang University, Urumqi 830046, China
[2] Xinjiang Key Laboratory of Solid State Physics and Devices, Xinjiang University, Urumqi 830046, China
* Correspondence: wuzf@xju.edu.cn (Z.W.); sunjun@xju.edu.cn (J.S.)

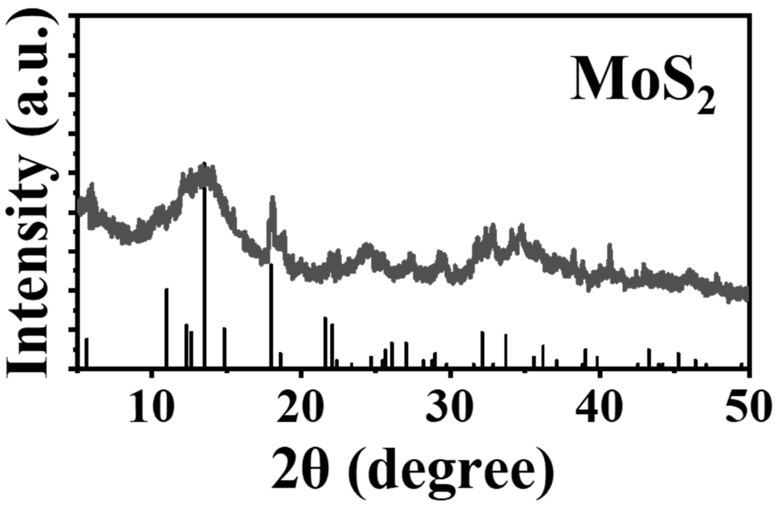

Figure S1. XRD patterns of MoS_2 with standard XRD JPCDS card of MoS_2.

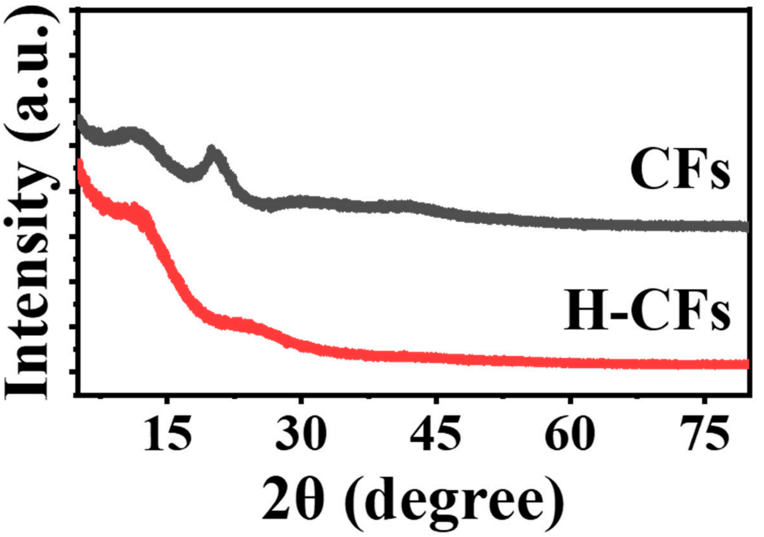

Figure S2. XRD patterns of CFs and H-CFs.

Citation: Zhang, Y.; Wu, Z.; Sun, J.; Sun, Q.; Chen, F.; Zhang, M.; Duan, H. Synthesis and Sensing Performance of Chitin Fiber/MoS_2 Composites. *Nanomaterials* **2023**, *13*, 1567. https://doi.org/10.3390/nano13091567

Academic Editor: Marco Cannas

Received: 4 April 2023
Revised: 26 April 2023
Accepted: 28 April 2023
Published: 6 May 2023

Copyright: © 2023 by the authors. Licensee MDPI, Basel, Switzerland. This article is an open access article distributed under the terms and conditions of the Creative Commons Attribution (CC BY) license (https://creativecommons.org/licenses/by/4.0/).

Table S1. Scanning analysis of C, N, O, S and Mo elements in EDS-Mapping.

**Type of element**	**Atomic %**	**Intensity (c/s)**	**Atomic Ratio**
C	37.550	1,102.90	1.0763
N	5.129	317.25	0.1470
O	34.888	2,273.73	1.0000
S	11.021	7,253.09	0.3159
Mo	11.412	6,841.62	0.3271
Total	100	--	--
